# Factors associated with mortality in patients with tuberculosis

**DOI:** 10.1186/1471-2334-10-258

**Published:** 2010-08-27

**Authors:** David J Horne, Rebecca Hubbard, Masahiro Narita, Alexia Exarchos, David R Park, Christopher H Goss

**Affiliations:** 1 Department of Medicine, University of Washington School of Medicine, Seattle, WA, USA; 2 Group Health Center for Health Studies, Seattle, WA, USA; 3 Department of Biostatistics, University of Washington School of Public Health, Seattle, WA, USA; 4 Department of Epidemiology, School of Public Health, University of Washington, Seattle, WA, USA; 5 Public Health - Seattle & King County, Tuberculosis Control Program, Seattle, WA, USA; 6 Washington State Department of Health, Infectious Disease and Reproductive Health Assessment Unit, Olympia, WA, USA

## Abstract

**Background:**

Known risk factors for death following a diagnosis of tuberculosis may not be applicable to current U.S. cases. We evaluated the factors associated with all-cause mortality in patients with tuberculosis in Washington State.

**Methods:**

Using data from the Tuberculosis Information Management System of Washington State, we conducted a cohort study of all residents diagnosed with tuberculosis from 1993 through 2005. Death from any cause was ascertained through the Washington State Death Certificate Data Files. Proportional hazards models were used to estimate the independent effect on all-cause mortality of demographic, clinical, and behavioral characteristics.

**Results:**

During a median follow-up of 6 years in 3451 patients treated for tuberculosis, there were 417 deaths. Mortality was independently associated with increasing age, male gender, HIV-coinfection, and U.S. birth. Within 1 year of tuberculosis diagnosis, treatment by a private provider and the use of directly observed therapy were also independently associated with increased mortality. In addition, an interaction term of private provider times directly observed therapy was also significantly associated with mortality.

**Conclusions:**

We identified factors independently associated with increased all-cause mortality following a diagnosis of tuberculosis. The associations between mortality and provider type should be evaluated with more thorough adjustment for severity of illness, but suggest important directions for future research.

## Background

There were over 1.7 million deaths due to tuberculosis (TB) worldwide in 2007 with a global case-fatality of 19% [1,2]. The United States TB-specific case-fatality has been estimated at 5% or less in recent years, although a Baltimore-based study observed a case-fatality of 24% in sputum smear positive patients while on treatment [2,3]. Studies evaluating risk factors for death following a diagnosis of TB have typically focused on specific patient populations (e.g. patients with multidrug resistant-TB (MDR-TB), human immunodeficiency virus (HIV)-coinfected patients, and patients enrolled in TB treatment trials) [4-8]. Population-based studies have identified a number of risk factors including HIV-coinfection, non-use of directly observed therapy (DOT), care from a physician inexperienced with TB management, drug resistance, and more than one site of TB involvement as associated with death following a diagnosis of TB [7,9-11].

The applicability of these prior studies to the U.S. is unknown as they were conducted outside of the United States or during the TB resurgence of the 1990s. The current characteristics of TB cases and TB control activities in the U.S. include increased use of DOT, increased proportion of TB due to foreign born persons, and greater delivery of TB treatment by public health departments; Washington State has mirrored these trends [12]. We performed a population-based study of factors associated with mortality following a diagnosis of TB in Washington State during a period of decreasing TB incidence and stable treatment recommendations [13]. We examined demographic, clinical and treatment related characteristics associated with survival. Some of the results of this study have been previously reported in the form of an abstract [14].

## Methods

### Study Population

The population in this cohort study consisted of all individuals diagnosed with active TB and reported to the Washington State Department of Health Tuberculosis Registry through the TB Information Management System (TIMS) during the period of January 1, 1993 through December 31, 2005. Tuberculosis is a reportable disease and the completeness of reporting in Washington State is estimated to be nearly 100 percent [15]. TB cases were defined according to the Centers for Disease Control and Prevention (CDC) guidelines [16]. The Washington State Institutional Review Board approved all study protocols and analyses.

### Data Collection

We merged data from two sources: 1) TIMS, and 2) the Washington State Death Certificate Data Files. The Report of Verified Case of TB (RVCT) is the national TB surveillance form produced by the CDC. Local public health jurisdictions in Washington State are required to complete and submit the RVCT to the State, where information is entered into TIMS. Data on deaths in Washington State and deaths of Washington State residents who died in other states are maintained in the Death Certificate Files. We obtained death registry information from January 1, 1993 to December 31, 2005. First and last names, as well as middle initials and home address when available, were used in conjunction with birth dates to link study subjects with data from the death registry.

Person-time of cohort members began when anti-tuberculosis treatment was started and ended on the date of death or December 31, 2005, whichever came first (information on the timing of the index culture was available only for patients with pulmonary TB). We decided on the variables for our model *a priori *based on known and potential risk factors, including: subject characteristics (age, gender, race/ethnicity, HIV status, U.S. birth, residence, substance use, income), disease characteristics (site of TB disease, radiographic cavitary disease, drug susceptibility, prior TB), and factors related to treatment (provider type, use of DOT). These factors were defined according to the RVCT [17]. Race and ethnicity were based on self-report. Provider type was defined as a health department if outpatient care was provided by a state or local health department. DOT was defined as treatment ingestion observed by a health care provider or other reliable person [17]. Variables available for the analysis that were not defined by the RVCT included recency of immigration to the United States and median household income. The latter was based on U.S. census data from 2000 using the subjects' home zip code. Extrapulmonary TB was defined as disease in organs other than the lungs or pleura [7]. Pulmonary TB with cavitation was defined as the presence of cavitary lesions on chest radiograph. In order to assess for temporal affects on survival, we included a variable based on diagnosis in the first half or last half of the study period ("year of diagnosis after 1999").

In the bivariate and multivariable analyses, we made a number of assumptions. Provider type was categorized as private provider if TB care was never received from a health department; otherwise care was categorized as a health department. For example, a subject who was discharged from a hospital and received treatment through a health department would be categorized as having a provider type of health department. Therapy type was categorized as self-administered or "other" (including DOT only or a combination of the two). Because of limited numbers, Pacific Islanders/Hawaiians were included in the Asian category. If information was missing regarding alcohol abuse, then patients were classified as non-abusers. Similar assumptions were made for injection drug use, homelessness and institutional residence. Subjects were considered HIV-positive only if they were known to be seropositive. Patients without drug susceptibility results were classified as having susceptible isolates.

### Statistical Analysis

We used Cox proportional hazards models to analyze associations between candidate risk factors and survival. The proportional hazards assumption was tested using graphical inspection and tests for time trends of Schoenfeld residuals. Variables that did not satisfy the proportional hazards assumption (income, health care provider type, and therapy type), were modelled after being stratified by time. We also examined the significance of two-way interactions between these time-stratified variables, in addition to interactions between age and sex. All of our pre-selected variables were included in the final multivariable models based on our initial hypotheses. To examine the effect of missing data and our assumptions on the model, we analyzed the data: 1) after excluding patients with missing data, and 2) imputing values for missing data based on frequencies observed for subjects with non-missing data. After observing the relationship between provider type and survival, we conducted a simulation study to assess the potential impact of differential loss to follow-up among subjects by provider type (see additional file 1). Statistical analyses were performed using Stata 10 (StataCorp, College Station, TX) and R 2.2.0 (R Foundation for Statistical Computing, Vienna, Austria).

## Results

During the 13-year study period, 3451 persons with active TB were diagnosed in Washington State. The median follow-up period was 5.9 years (interquartile range [IQR]: 2.6-9.3). There were 417 deaths and cumulative all-cause mortality at years 1 and 3 were 4.6% (95% confidence interval [CI] 3.9-5.4) and 7.7% (CI 6.8-8.8), respectively. Among HIV-coinfected patients the one-year and three-year mortality were 10.5% (CI 6.4-17.2) and 18.9% (CI 12.8-27.8), respectively.

The mean (± SD) age at the time of diagnosis was 45 (± 21) years. The date of initial sputum culture positivity was reported in 1568 patients (68% of the pulmonary TB cases), and anti-tuberculosis therapy was started less than one week after collection of the initial sputum culture in 70%. The median time to treatment initiation in these 1568 patients was 2 days after sputum collection (IQR: 0-12). In the entire cohort, 85% of patients were documented as completing therapy, 3% moved prior to completion of treatment, 2% of patients were considered lost and 1% refused treatment; among the remaining patients, 5% died, 1% had "other" as the stop reason, and information was missing in 3%.

Baseline characteristics for the cohort are in Table 1. Most subjects were male (61%), foreign born (63%), received DOT (61%), and were treated exclusively by a public health department (54%). There was a low prevalence of known HIV-coinfection (5%). Except for resistance to isoniazid (8%) and streptomycin (6.7%), there was less than 2% prevalence of resistance to any single drug; the prevalence of MDR-TB was 1%. Mortality was lowest in females and in persons who were younger, non-Caucasian, HIV-negative, and foreign born, and also in persons without a prior history of TB or alcohol abuse. The presence of both pulmonary and extrapulmonary diseases was associated with an elevated death rate, as was receipt of care through a private provider.

**Table 1:** Person-years, deaths and death rates by selected baseline characteristics

Characteristics	N (%) (total = 3451)	Person- years	Mortality Rate	95% CI
Age, years: median (SD)	45 (21)			
Gender				
Female	1349 (39.1)	8183	12.3	10.2-15.0
Male	2102 (60.9)	12585	21.7	19.3-24.4
Race				
Asian/Pacific Islander	1321 (38.2)	8159	10.3	8.3-12.8
Black	438 (12.7)	2316	13.4	9.4-19.0
Caucasian	982 (28.5)	5859	35.2	30.7-40.3
Latino	502 (14.5)	3097	7.8	5.2-11.6
Native American	179 (5.2)	1093	23.8	16.2-34.9
Unknown	29 (0.8)	204	14.7	4.7-45.6
HIV Status				
Negative	1976 (57.2)	11753	11.7	9.9-13.8
Positive	166 (4.8)	905	44.2	32.4-60.2
Unknown	1309 (37.9)	8109	24.2	21.1-27.9
Major site of disease				
Pulmonary/pleural	2652 (76.8)	16134	18.6	16.6-20.8
Lymphatic	425 (12.3)	2459	11.0	7.5-16.0
Bone-joint/GU/Peritoneal	206 (5.9)	1264	19.8	13.4-29.3
Miliary/Meningeal	92 (2.7)	515	33.0	20.5-53.1
Other	76 (2.2)	395	5.3	5.3-30.4
Site of disease				
Pulmonary	2288 (66.3)	14229	18.3	16.2-20.6
Extrapulmonary	837 (24.2)	4887	13.3	10.4-17.0
Both	326 (9.4)	1652	29.7	22.4-39.3
Sputum Smear				
Positive	1218 (35.3)	7150	20.6	17.5-24.2
Negative	1290 (37.4)	7426	16.3	13.6-19.5
Not applicable/missing	943 (27.3)	6191	17.1	14.2-20.7
Chest x-ray				
Cavitary	626 (18.1)	3769	15.1	11.7-19.6
Non-cavitary	2237 (64.8)	13107	20.0	17.7-22.6
Unknown	588 (17.0)	3891	14.1	10.9-18.4
Isoniazid susceptibility				
Resistant	281 (8.1)	1713	11.7	7.5-18.1
Susceptible	2529 (73.3)	14856	20.1	17.9-22.5
Unknown	641 (18.6)	4199	13.3	10.3-17.3
Streptomycin susceptibility				
Resistant	241 (7.0)	1432	7.0	3.8-13.0
Susceptible	2530 (73.3)	15062	20.2	18.0-22.6
Unknown	680 (19.7)	4274	14.0	10.9-18.1
Previous TB				
No	3168 (91.8)	19245	17.3	15.5-19.2
Yes	211 (6.1)	1210	27.3	19.4-38.4
Unknown	72 (2.1)	313	28.8	15.0-55.3
Excess alcohol use				
No	2924 (84.7)	17558	16.3	14.6-18.4
Yes	346 (10.0)	1849	29.7	22.8-38.7
Unknown	181 (5.2)	1360	23.5	16.6-33.3
Injection drug use				
No	3090 (89.5)	19108	17.5	15.8-19.5
Yes	170 (4.9)	395	32.9	19.1-56.7
Unknown	191 (5.5)	1264	20.6	14.0-30.2
Correctional facility				
No	3373 (97.7)	20240	18.1	16.3-20.0
Yes	93 (2.7)	397	15.1	6.8-33.6
Homeless				
No	3073 (89.0)	18790	17.0	15.3-19.0
Yes	352 (10.2)	1788	28.0	21.2-36.9
Unknown	26 (0.8)	170	17.7	5.7-54.7
Long-term care				
No	3358 (97.3)	20341	17.1	15.4-19.0
Yes	93 (2.7)	397	65.2	44.4-95.8
Therapy type				
Directly observed therapy	2116 (61.3)	11794	19.2	16.8-21.8
Self-administered therapy	854 (24.7)	6596	17.7	14.8-21.3
Both	391 (11.3)	1977	11.6	7.7-17.5
Unknown	90 (2.6)	401	20.0	10.0-39.9
Health care provider type				
Health department	1864 (54.0)	11727	10.3	8.6-12.3
Private	656 (19.0)	4081	32.6	27.5-38.6
Both	880 (25.5)	4681	24.4	20.3-29.3
Unknown	51 (1.5)	206	21.5	9.7-47.9
Immigration status				
U.S.-born	1283 (37.2)	7546	34.5	30.5-38.9
Foreign-born	2168 (62.8)			
U.S. resident ≥2 years	1208 (55.7)	14332	24.5	22.1-27.2
U.S. resident <2 years	960 (44.3)	6436	3.6	2.4-5.4

In the multivariable model that included all hypothesized risk factors, we found that increasing age, male gender, HIV-coinfection, treatment by a private health care provider, the use of DOT, U.S. birth, and not being a recent immigrant were independently associated with increased mortality (Table 2). In developing our Cox proportional hazards model, income, health care provider type, and therapy type did not satisfy assumptions of proportional hazards. However, after stratifying by before or after the first year of follow-up, these variables satisfied the proportional hazards assumption. Treatment by a private health care provider (HR 5.1; 95% CI, 3.5-7.3) and use of DOT (HR 3.0; 95% CI, 1.9-4.5) were associated with death only within one year of the diagnosis of TB; patients who survived one year were no longer at increased risk of death based on these variables. Income, after time stratification, was no longer associated with survival. There were no significant interactions between gender and age.

**Table 2:** All-cause mortality using proportional hazards model including time stratified effects for income, provider, and therapy

Variable	HR	95% CI	p
Age (year)	1.05	1.05, 1.06	<0.001
Male	1.5	1.2, 2.0	0.001
Race/ethnicity			
Asian	Reference	-------	------
Black	0.7	0.4, 1.2	0.22
Hispanic	0.9	0.5, 1.4	0.56
Native American	1.1	0.6, 2.0	0.70
White	1.2	0.8, 1.8	0.45
U.S. Born	1.7	1.2, 2.5	0.008
Foreign-born, U.S. resident <2 years*	0.4	0.3, 0.7	<0.001
HIV-positive†	4.2	2.8, 6.2	<0.001
Major site of Disease			
Pulmonary	Reference	-------	------
Lymphatic	1.0	0.6, 1.5	0.82
Bone or joint/Genitourinary/Peritoneal	1.1	0.7, 1.7	0.62
Miliary/meningeal	1.3	0.8, 2.1	0.31
Other	0.7	0.3, 1.7	0.48
Radiographic cavitary disease	1.1	0.8, 1.6	0.44
Drug susceptibility||			
INH susceptible	1.0	0.6, 1.7	0.99
Streptomycin susceptible	1.7	0.8, 3.7	0.18
Previous history of TB	1.0	0.7, 1.6	0.84
Alcohol abuse	1.2	0.8, 1.7	0.41
Intravenous drug use	1.4	0.8, 2.2	0.24
Homelessness	0.9	0.6, 1.2	0.41
Long-term care resident	1.1	0.7, 1.7	0.76
Income ($5000)			
First year	0.9	0.9, 1.0	0.05
After first year	1.0	0.9, 1.0	0.25
Private provider only			
Within 1 year of TB diagnosis	5.1	3.5, 7.3	<0.001
More than 1 year after TB diagnosis	1.1	0.7, 1.5	0.77
Directly observed therapy			
Within 1 year of TB diagnosis	3.0	1.9, 4.5	<0.001
More than 1 year after TB diagnosis	1.0	0.7, 1.4	0.94
Year of diagnosis after 1999	1.0	0.8, 1.3	0.97

*Reference is immigration more than 2 years prior to TB diagnosis

†Reference category is HIV-negative or unknown

||Compared to INH and streptomycin susceptible isolates, respectively. Other resistance patterns not included in the model due to limited numbers of isolates.

We evaluated our time stratified variables by modelling an interaction between health care provider type and therapy type (Table 3). Different from our final model, we found that receiving treatment from a private health care provider was significantly associated with decreased survival during the first year after a diagnosis of TB regardless of therapy type, although the strength of the association was greater if DOT was used (HR 8.5; 95% CI, 4.3-17.1) than if treatment was solely self-administered (HR 2.2; 95% CI, 1.1-4.6).

**Table 3:** All-cause mortality using proportional hazards model including directly observed therapy and provider type interaction terms

Variable	HR	95% CI
Age	1.05	1.05, 1.06
Male	1.5	1.2, 2.0
HIV-positive	4.2	2.9, 6.3
Provider type × DOT (≤1 year)*		
Private provider, no DOT	2.2	1.1, 4.6
Health department, DOT	1.4	0.7, 2.8
Private provider, DOT	8.5	4.3, 17.1
Provider type × DOT (>1 year)*		
Private provider, no DOT	1.1	0.7, 1.7
Health department, DOT	1.0	0.7, 1.5
Private provider, DOT	1.0	0.5, 2.0
Recent immigrant	0.4	0.3, 0.7
Not foreign born	1.7	1.2, 2.5

* Reference group is health department or both, no DOT

Except for interaction results, only statistically significant (p < 0.05) effects are reported. The model was additionally adjusted for race, income, major site of disease, INH susceptibility, streptomycin susceptibility, previous TB, excess alcohol use, excess drug use, homelessness, in long term care, cavitary disease, and year of diagnosis before or after 1999.

TB-related deaths were 21% of all deaths and 39% of the deaths that occurred within 12 months of starting TB treatment. We assessed the effect on the model of limiting mortality to TB-related deaths, defined as a TB-related International Classification of Diseases code listed under the multiple contributing causes of death on the death certificate. Increasing age and HIV-coinfection were associated with a TB-related death; within one year of TB treatment initiation, treatment by a private health care provider and use of DOT were also associated with a TB-related death (see additional file 2).

As differences in illness severity could introduce confounding if a subject's death and TB diagnosis occurred during the same hospitalization, we assessed the effects on our model of excluding subjects who died within 30-days of a diagnosis of TB (n = 92). Although the HR associated with treatment by a private health care provider was attenuated, it remained elevated (HR 2.8, 1.7-4.6; see additional file 3). In order to assess the impact of missing data and our assumptions on our models, we repeated our analyses after: 1) excluding patients with missing data, and 2) using imputed values for missing data. There were no significant changes in the observed associations compared to our final model (results not shown).

We evaluated the association of sputum smear positivity and survival in patients with pulmonary TB. Neither sputum smear positivity nor cavitation on chest radiographs was found to be associated with survival (see additional file 4). In patients with date of initial culture collection, a delay in treatment (defined as treatment initiation more than one week after sputum collection) was not associated with death in bivariate or multivariable analysis.

## Discussion

All-cause mortality in our study was predicted by older age, HIV-coinfection, male gender, U.S. birth, remote immigration, care through a private health care provider and the use of DOT. The association between care through a private provider and the use of DOT was present only for the 12 month period following TB treatment initiation.

Age, HIV-coinfection and male gender have been previously identified as risk factors for death in subjects diagnosed with TB [7]. To our knowledge, U.S. birth, and recent immigration have not been previously described in relation to survival. U.S. birth has previously been reported as associated with relapse and recurrence of TB [18]. In general, longer life expectancy among immigrant groups has been observed in many types of epidemiologic studies and may relate to a "healthy immigrant effect" or healthier behaviors after immigration compared to U.S -born individuals [19]. A measure of disease severity, site of TB involvement (i.e. miliary/meningeal), was associated with poorer survival in bivariate analysis but did not remain so after adjustment for HIV status.

We found an association of decreased survival and TB treatment from a private provider during the first year following treatment initiation for TB and this relationship remained when evaluating only those patients that experienced a TB related death. In our model, an individual who received TB treatment from a private provider and survived one year would no longer be at increased risk of death based on this variable. An association between increased physician experience in treating TB and improved survival was recently reported in a Canadian study [11]. Previous studies have observed that management of TB by non-public health providers is associated with inappropriate treatment regimens and lower likelihood of cure. (24, 25) In our cohort, one indicator of quality of TB care, documentation of sputum culture conversion in patients with pulmonary TB that completed treatment differed between health department (93%) and private providers (67%); similarly HIV status was assessed in 64% of patients treated by a health department and 52% of patients treated by private providers (p-value for both < 0.001).

DOT has been associated with improved likelihood of completion of therapy, decreased relapse, and decreased mortality [11,20-22]. However, a recent study from Spain also found an association between the use of DOT and increased mortality; the authors hypothesized that DOT may have been preferentially used in complicated patients [23]. We modelled an interaction between health care provider type and therapy type to evaluate our findings. The use of DOT by health departments in this model was not significantly associated with differences in survival. However, we did find an association between TB medical care by private physicians and decreased survival regardless of the therapy type (i.e. DOT or self-administered), although this association is much stronger in patients who received DOT or combination therapy. A potential explanation for this may be an indication bias where DOT is utilized by private providers based on increased severity of illness or decreased social support. In bivariate analyses, we found increased use of DOT among patients with smear-positive sputum (84% vs. 78%, p-value 0.001), cavitary disease (83% vs. 78%, p-value 0.005), the homeless (96% vs. 72%, p-value < 0.001) and lower incomes (p < 0.001).

There are limitations to our study. First, we were limited in our ability to address potential confounding, particularly related to differences in severity of illness. The observed association between health care provider type and survival could be confounded by differences in severity of illness. We attempted to grade TB severity by major organ site of involvement and the presence of radiographic cavitations. In addition, we performed a sensitivity analysis to address potential confounding due to differences in severity of illness by excluding deaths within 30 days of TB treatment initiation. Second, survival follow-up was passive and our results could also be influenced by incomplete ascertainment of deaths. A disproportionate loss of subjects following the completion of treatment for TB who received their care through a public health department could create the appearance of decreased survival among subjects treated by private health care providers. We simulated plausible differences in follow-up to assess how this would induce an artificial association with survival. Our results suggest that it is extremely unlikely that the hazard ratio we observed was due in any large part to differential loss to follow-up. (see additional file 1). Finally, our study was based in a single state in which there is a low prevalence of multidrug-resistant TB and may not apply to other regions with higher prevalence of drug resistance.

Despite these limitations, we believe that this study has important strengths. It is a population-based study that reflects nationwide TB trends. Although we used all-cause mortality as the primary outcome of our analysis, similar to other studies of survival following a diagnosis of TB, our observed associations persisted when we limited the outcome to TB related deaths [7,11,24]. Our findings, particularly related to associations between provider type and survival, need to be verified in studies where potential confounders can be assessed. If the findings are verified, this would support further efforts to identify mechanisms for the observed differences and work to eliminate disparities in survival. Finally, it is important to emphasize that our findings regarding the use of DOT are likely due to differences in the types of patients recommended for DOT in our study population.

## Conclusions

In conclusion, we identified associations between decreased survival in TB patients and age, male gender, HIV-coinfection, US birth, remote immigration, DOT, and TB treatment by a private health care provider. Although it is important to acknowledge the potential for unmeasured confounding, our study suggests that additional studies are needed to assess associations between TB-related physician experience and TB treatment outcomes.

## Competing interests

The authors declare that they have no competing interests.

## Authors' contributions

DJH participated in study design, data acquisition, statistical analysis, data interpretation, drafting of the manuscript, and critical revision of the manuscript. RH participated in statistical analysis, data interpretation, drafting of the manuscript, and critical revision of the manuscript. MN participated in data acquisition and critical revision of the manuscript. AE participated in data acquisition, data interpretation, and critical revision of the manuscript. DRP participated in study design and critical revision of the manuscript. CHG participated in study design, statistical analysis, data interpretation, drafting of the manuscript, and critical revision of the manuscript. All authors read and approved the final manuscript.

## Pre-publication history

The pre-publication history for this paper can be accessed here:

http://www.biomedcentral.com/1471-2334/10/258/prepub

## Supplementary Material

Additional file 1 **Differential loss to follow-up simulation**. A simulation study to assess the potential impact of differential loss to follow-up among subjects treated by private providers as compared to health departments. Click here for file

Additional file 2 **Tuberculosis-related mortality using proportional hazards model including time stratified effects for income, provider, and therapy**. We assessed the effect on the model of limiting mortality to TB-related deaths, defined as a TB-related International Classification of Diseases code listed under the multiple contributing causes of death on the death certificate. Click here for file

Additional file 3 **Results of sensitivity analysis after excluding subjects who died within the first 30 days**. As differences in illness severity could introduce confounding if a subject's death and TB diagnosis occurred during the same hospitalization, we assessed the effects on our model of excluding subjects who died within 30-days of a diagnosis of TB. Click here for file

Additional file 4 **Results of analysis evaluating an association between sputum smear status and survival**. We evaluated the association of sputum smear positivity and survival in patients with pulmonary TB. Click here for file
