# Supplementary material for: Factors associated with mortality in patients with tuberculosis
Source: BMC Infect Dis. 2010 Aug 27;10:258. doi: 10.1186/1471-2334-10-258 (PMC2936899; doi:10.1186/1471-2334-10-258)
Supplement: Additional file 1 — Differential loss to follow-up simulation. A simulation study to assess the potential impact of differential loss to follow-up among subjects treated by private providers as compared to health departments. [file 1471-2334-10-258-S1.DOCX]

### Additional file 1

*Differential loss to follow-up simulation*

We conducted a simulation study to assess the potential impact of differential loss to follow-up among subjects treated by private providers as compared to health departments. Data were simulated using death rates and distributions of death and censoring times based on those seen in the data. Provider type was simulated at the rate seen in the real data and assuming there was no relationship between provider type and time to death. Therefore, in these simulations the true hazard ratio for provider type is 1. We introduced loss to follow-up with subjects lost to follow-up with probability of loss dependent on provider type. Subjects lost to follow-up were censored and given the maximum time until censoring. We fit a Cox proportional hazards model for the effect of provider type on hazard of death to simulated data. The probability of loss to follow-up in each provider type was varied 0.005 to 0.5. 100 simulated data sets were generated for each probability of loss to follow-up.

Differential loss to follow-up induces a relationship between provider type and survival. The increased hazard ratio induced by differential loss to follow-up is highest for low rates of loss to follow-up in private providers. However, even for loss to follow-up rate of 50% among health departments, the induced hazard ratio is about 2.5, half what was observed in the real data. This suggests that it is extremely unlikely that the hazard ratio we have observed in these data was be due to differential loss to follow-up.
