# Supplementary material for: Factors associated with mortality in patients with tuberculosis
Source: BMC Infect Dis. 2010 Aug 27;10:258. doi: 10.1186/1471-2334-10-258 (PMC2936899; doi:10.1186/1471-2334-10-258)
Supplement: Additional file 2 — Tuberculosis-related mortality using proportional hazards model including time stratified effects for income, provider, and therapy. We assessed the effect on the model of limiting mortality to TB-related deaths, defined as a TB-related International Classification of Diseases code listed under the multiple contributing causes of death on the death certificate. [file 1471-2334-10-258-S2.DOC]

### Additional file 2

### Table - Tuberculosis-related mortality using proportional hazards model including time stratified effects for income, provider, and therapy

| Variable | | HR | | 95% CI |
| --- | --- | --- | --- | --- |
| Age | 1.04 | | 1.03, 1.06 | |
| HIV-positive | 2.9 | | 1.1, 7.9 | |
| Private provider only |  | |  | |
| Within 1 year of TB diagnosis | 8.7 | | 5.0, 14.9 | |
| More than 1 year after TB diagnosis | 3.7 | | 0.9, 15.4 | |
| Directly observed therapy |  | |  | |
| Within 1 year of TB diagnosis | 5.6 | | 2.8, 11.4 | |
| More than 1 year after TB diagnosis | 1.8 | | 0.4, 8.0 | |
| Foreign-born, U.S. resident < 2 years | 0.2 | | 0.1, 0.6 | |

The model was additionally adjusted for gender, income, race, major site of disease, INH susceptibility, streptomycin susceptibility, previous TB, excess alcohol use, excess drug use, cavitary disease, U.S. birth, and year of diagnosis before or after 1999.
