# Supplementary material for: Factors associated with mortality in patients with tuberculosis
Source: BMC Infect Dis. 2010 Aug 27;10:258. doi: 10.1186/1471-2334-10-258 (PMC2936899; doi:10.1186/1471-2334-10-258)
Supplement: Additional file 3 — Results of sensitivity analysis after excluding subjects who died within the first 30 days. As differences in illness severity could introduce confounding if a subject's death and TB diagnosis occurred during the same hospitalization, we assessed the effects on our model of excluding subjects who died within 30-days of a diagnosis of TB. [file 1471-2334-10-258-S3.DOCX]

### Additional file 3

### Table - Proportional hazards model excluding subjects with less than 30 days of survival.

| Variable | | HR | | 95% CI | |
| --- | --- | --- | --- | --- | --- |
| Age | | 1.06 | | 1.05, 1.07 |  |
| Male | | 1.6 | | 1.2, 2.1 |  |
| HIV Positive | | 4.2 | | 2.7, 6.3 |  |
| Private provider only | |  | |  |  |
| First year | | 2.8 | | 1.7, 4.5 |  |
| After first year | | 1.0 | | 0.7, 1.5 |  |
| Directly observed therapy | |  | |  |  |
| First year | | 2.7 | | 1.6, 4.7 |  |
| After first year | | 1.1 | | 0.7, 1.5 |  |
| Recent immigrant | | 0.5 | | 0.3, 0.8 |  |
| Not foreign born | | 2.0 | | 1.3, 3.2 |  |

Only statistically significant (p < 0.05) effects are reported. The model was additionally adjusted for race, income, major site of disease, INH susceptibility, streptomycin susceptibility, previous TB, excess alcohol use, excess drug use, homelessness, in long term care, cavitary disease, and year of diagnosis before or after 1999.
