# Supplementary material for: Factors associated with mortality in patients with tuberculosis
Source: BMC Infect Dis. 2010 Aug 27;10:258. doi: 10.1186/1471-2334-10-258 (PMC2936899; doi:10.1186/1471-2334-10-258)
Supplement: Additional file 4 — Results of analysis evaluating an association between sputum smear status and survival. We evaluated the association of sputum smear positivity and survival in patients with pulmonary TB. [file 1471-2334-10-258-S4.DOCX]

### Additional file 4

### Table - Proportional hazards model for subjects with pulmonary TB only.

| Variable | | HR | 95% CI | |
| --- | --- | --- | --- | --- |
| Age | 1.05 | | 1.04, 1.06 |  |
| Male | 1.4 | | 1.03, 1.9 |  |
| HIV Positive | 2.9 | | 1.8, 4.7 |  |
| Private provider only |  | |  |  |
| First year | 6.2 | | 4.0, 9.7 |  |
| After first year | 0.8 | | 0.5, 1.4 |  |
| Directly observed therapy |  | |  |  |
| First year | 2.9 | | 1.6, 5.0 |  |
| After first year | 0.9 | | 0.6, 1.3 |  |
| Recent immigrant | 0.4 | | 0.3, 0.8 |  |
| Not foreign born | 1.9 | | 1.1, 3.1 |  |

Only statistically significant (p < 0.05) effects are reported. The model was additionally adjusted for race, income, sputum smear negative, INH susceptibility, previous TB, excess alcohol use, excess drug use, homelessness, in long term care, cavitary disease, and year of diagnosis before or after 1999.
